# Presenteeism Among Healthcare Providers, Staff, and Students in Jalisco, Mexico: A Descriptive Study

**DOI:** 10.7759/cureus.28533

**Published:** 2022-08-29

**Authors:** Eric C Shattuck, Igor M Ramos Herrera, Thankam Sunil

**Affiliations:** 1 Institute for Health Disparities Research, University of Texas at San Antonio, San Antonio, USA; 2 Public Health, University of Texas at San Antonio, San Antonio, USA; 3 Public Health, University of Guadalajara, Guadalajara, MEX; 4 Public Health, The University of Tennessee, Knoxville, USA

**Keywords:** nosocomial outbreaks, mexico, infectious disease transmission, healthcare workers, presenteeism

## Abstract

Objective

We measured presenteeism (continuing to attend work or other activities while sick) in a sample of healthcare workers in Jalisco, Mexico to better understand the phenomenon, which can place patients at risk of infection.

Methods

An online survey link was distributed to all healthcare professionals, staff, and students registered with the Jalisco Ministry of Health starting in March 2020. Completed surveys (n = 196) collected between March and July 2020 were analyzed using bivariate and descriptive statistics including Kruskal-Wallis rank sum tests and Fisher’s tests.

Results

Most participants (67.5%) reported working while sick. Primary reasons included concerns about patients and continuity of care. Approximately 97% of respondents believed that working while sick could put patients at risk but still attended work with multiple symptoms.

Conclusion

These presenteeism rates and motivations are comparable to data from the US and other countries. We suggest that state and federal medical organizations address presenteeism to prevent nosocomial outbreaks.

## Introduction

Presenteeism, or continuing to attend work, school, or other normal activities while sick, is associated with multiple negative outcomes [1]. These include more errors at work, lower productivity, and negative health impacts [2-4]. While presenteeism is often discussed in terms of chronic health conditions, a recent review by Webster and co-authors has shown that presenteeism related to infectious disease is common, with an overall prevalence of 35-97% [5]. Risks here include possible pathogen transmission from sick to healthy workers, reduced productivity, and potentially a longer course of illness or complications in sick workers forgoing rest and recuperation. These risks are magnified in healthcare settings, where doctors, nurses, and other staff could transmit pathogens to immunocompromised or otherwise vulnerable patients. Indeed, an outbreak of influenza A in an oncology ward has been tentatively linked to a sick healthcare worker [6]. Infection control measures included closing the ward, prophylactic oseltamivir treatment, and temporary dismissal of symptomatic healthcare workers, resulting in expenses in the tens of thousands of dollars for testing and treatment, loss of highly specialized manpower, and a limited ability to provide care to current or new oncology patients [6].

Rates of presenteeism in healthcare settings (37-97%) mirror those in the general population [6]. Surveys of healthcare workers (HCWs) have highlighted many potential reasons for presenteeism. The predominant reason in the case of the influenza A outbreak discussed above was a “sense of duty as a healthcare worker” followed by “viewed illness as too minor to pose a risk to others” [6]. In their survey of over 500 healthcare professionals, Szymczak and colleagues found that “not wanting to let colleagues down,” staffing concerns, and “not wanting to let patients down” were the top three reported reasons for presenteeism [7]. There were also significant differences between job types. Advance practice clinicians were more concerned with being ostracized by their colleagues if they did not attend work while sick compared to attending physicians, who were more concerned about continuity of care [7].

Another major reason identified in this survey is uncertainty about what constitutes “too sick to work.” In a multinational sample of both HCWs and non-HCWs, Tartari and colleagues found that an overwhelming majority of both groups would continue to work while suffering minor influenza-like illness (ILI) symptoms, including fatigue, sneezing, and cough. While there was a general drop in numbers of people who reported working with more severe ILI symptoms (e.g., cold/chills, fever, and others), numbers were still higher than ideal, including 27% of HCWs who reported working with a fever, contrary to the US Centers for Disease Control and Prevention’s (CDC) guidance [8]. In this same study, when asked which combination of ILI symptoms would keep them home and which symptoms were most relevant, participants reported nearly 200 unique combinations. This suggests considerable disagreement regarding which symptoms constitute severe and minor/mild ILI and that greater clarity is needed surrounding symptom severity sufficient to preclude working for HCWs [8]. Similarly, Szymczak et al.’s survey found that most respondents would work with the acute onset of significant respiratory symptoms (56%) with diarrhea (30%) being the next most common acceptable symptom [7].

Research on HCW presenteeism highlights the complexities and uncertainties that go into individual decisions about working while sick, including aspects related to organizational cultures such as a sense of duty as a healthcare worker to continue to attend to patients while sick. However, most of the research on HCW presenteeism has been conducted in high-income countries in the global north. While Tartari and colleagues collected data from multiple countries, only about 10% of their sample were from middle- or low-income countries where constraints on staffing or paid sick leave may affect the landscape of presenteeism, and data were not disaggregated by global region or national income level [8]. We present results from a recent survey of healthcare workers from the Mexican state of Jalisco to both compare results to those from the global north and to increase our knowledge of presenteeism across international contexts.

## Materials and methods

A survey with multiple close-ended items and scales related to both burnout and presenteeism was electronically distributed to all healthcare workers (including administrative staff and medical students) in the Mexican state of Jalisco beginning in March 2020. Jalisco is the 7th largest state in Mexico with a population circa 2020 of roughly 8 million. Most of the population (approximately 5 million) is centered in the Guadalajara metropolitan area, making it the third most populous metropolitan area in the country.

Our survey was open to all healthcare workers in Jalisco. There were no exclusionary criteria. The research protocol and survey were approved by the University of Texas at San Antonio IRB (#20-064E) and all subjects gave their informed consent. The survey was translated into Spanish independently and cross-verified by two native speakers. The survey was hosted on the Qualtrics platform and distributed to healthcare workers by the Jalisco Ministry of Health through their email lists.

The first case of COVID-19 was reported in Jalisco on March 14th, 2020. A total of 340 responses were collected by July 2020. The survey was distributed to approximately 1,100 healthcare professionals in Jalisco (Dr. Alfonso Gutiérrez-Padilla, pers. comm.), giving a response rate of 31%. A recent meta-analysis of over 1000 reported online survey response rates found an average rate of 44% [9]. Our lower response rate may be attributable to the social effects of the COVID-19 pandemic, including shutdowns and increased demands on healthcare workers. Thirty-four responses in our survey were removed because they were only partially completed, resulting in 306 responses. A further 44 responses were dropped because they did not indicate a job type, while 66 responses were removed because they did not indicate whether they had ever worked while sick or not. Therefore, 196 complete responses were used for analysis.

 Two questions measured presenteeism: the first asked participants if they had ever gone to work when it was better to skip work due to sickness, while the second asked the participants how many times they had done this in the past year (1-5 or more times). To assess the types of symptoms healthcare workers would bring to work, we used the same list used by Szymczak and colleagues [7]. This included fever, aches, and chills (combined), vomiting only, respiratory symptoms, fever only, and others. Following Szymczak et al., we also asked participants to rank the relative importance of multiple possible drivers of presenteeism from 1 (not important) to 5 (extremely important) [7]. These included items such as, “I do not want to let my patients down,” “I am the only person who can carry out a particular task,” and “I come to work because my colleagues work while sick.” We also asked which behaviors participants took to help prevent transmission to patients and colleagues on those occasions when they came to work sick. Items included wearing a mask, wearing gloves, and avoiding immunocompromised patients. Finally, we asked whether participants believe that coming to work while sick puts patients at risk. Importantly, while Mexican HCWs began a reduced workload during the COVID-19 pandemic and frequently worked from home, the questions on our survey were related to general behaviors, e.g., “have you ever worked when sick”, or behaviors in the past year rather than behaviors since the start of the pandemic.

Descriptive statistics including means, value ranges, and standard deviations, were used to explore presenteeism in this sample. The normality of continuous data was determined by Shapiro-Wilk tests. Fisher’s tests for categorical data and two-tailed Kruskal-Wallis rank sum tests for continuous data were used to examine differences in presenteeism between job types (e.g., healthcare professionals vs. students). All analyses were conducted in R (v.3.6.3) and statistical significance was set at p ≤ 0.05 [10].

## Results

Mean age of the sample was 35.68 (sd = 12.86, range 23-77), 123 (62.76%) were healthcare professionals, 32 (16.33%) were administrators, 35 (17.86%) were students, and 4 (6%) were “other.” Basic descriptive statistics are shown in Table 1.

**Table 1:** Descriptive Statistics

	Healthcare Professionals (N=123)	Administrators (N=32)	Students (N=35)	Other (N=6)	Overall (N=196)
Age					
Mean (SD)	35.8 (12.2)	47.5 (11.5)	24.3 (1.47)	36.5 (14.5)	35.7 (12.9)
Min - Max	23 - 67	25 - 77	23 - 30	25 - 59	23.0 - 77
Missing	1 (0.8%)	0 (0%)	0 (0%)	0 (0%)	1 (0.5%)
Time In Current Position					
Less Than One Year	26 (21.1%)	1 (3.1%)	32 (91.4%)	3 (50.0%)	62 (31.6%)
1-10 Years	62 (50.4%)	12 (37.5%)	2 (5.7%)	2 (33.3%)	78 (39.8%)
More Than 10 Years	34 (27.6%)	19 (59.4%)	1 (2.9%)	1 (16.7%)	55 (28.1%)
Missing	1 (0.8%)	0 (0%)	0 (0%)	0 (0%)	1 (0.5%)

Eighty-three (67.5%) healthcare professionals reported that they had ever worked when sick. The rate of presenteeism was larger in other job types, with 24 (75%) of administrators, 29 (82.9%) of students, and 5 (83.3%) of other staff reporting presenteeism. Most individuals in each job category reported working while sick once or twice in the past year (Table 2). We found no relationship between job type and presenteeism (p = 0.306). After dropping “Other” professionals due to small cell sizes and a further 6 cases that did not answer the presenteeism frequency question, we found no relationship between job type and frequency of presenteeism (p = 0.382).

**Table 2:** Presenteeism frequency by job type

Frequency in past year	Professional	Admin.	Student	Other
Once	37 (44.6%)	14 (58.3%)	10 (34.5%)	3 (60.0%)
Twice	17 (20.5%)	2 (8.3%)	10 (34.5%)	1 (20.0%)
3 times	10 (12.0%)	2 (8.3%)	4 (13.8%)	0 (0.0%)
4 times	5 (6.0%)	0 (0.0%)	1 (3.4%)	0 (0.0%)
5+ times	11 (13.3%)	5 (20.8%)	3 (10.3%)	0 (0.0%)
N/A	3 (3.6%)	1 (4.2%)	1 (3.4%)	1 (20.0%)

Regarding reasons behind presenteeism, mean values were greatest for not wanting to let patients down (3.62), concerns about continuity of care (3.36), not enough available staff (3.53), and not wanting to let colleagues down (3.30). The least important were insufficient leave (0.67), fears of ostracism (1.32), and mimicking colleagues’ behavior (1.71). Using Kruskal-Wallis rank sum tests, we found significant relationships between job type (after dropping “Other”) and not wanting to let colleagues down (𝜒-sq. = 8.05, df = 2, p = 0.018), insufficient staff (𝜒-sq. = 6.25, df = 2, p = 0.044), and being the only qualified staff member (𝜒-sq. = 7.16, df = 2, p = 0.028). Post-hoc pairwise Mann-Whitney U tests with Bonferroni correction found a significant difference between healthcare professionals and administrators in not wanting to let colleagues down (3.57 vs. 2.48, respectively; p = 0.023) and feeling that one is the only qualified staff member (1.59 vs. 2.87, p = 0.023). Additionally, there was a borderline significant difference between healthcare professionals and administrators in the importance of not having sufficient staff (3.69 vs. 2.52; p = 0.054).

Congestion (n = 80), sore throat (n = 80), and cough (n = 78) were the symptoms most likely to be associated with presenteeism in the sample. Vomiting only (n = 28), fever, aches, and chills with diarrhea (n = 28), and fever, aches, and chills with vomiting (n = 18) were the least represented. Other important symptoms indicative of an infectious disease including respiratory symptoms (n = 55) and fever, aches, and chills (n = 41) were represented to varying degrees. Significant differences by job type were found for sore throat (p = 0.017), cough (p = 0.015), cough and rhinorrhea (p = 0.007), diarrhea (p = 0.004), vomiting (p = 0.021), and congestion (p = 0.007). Counts are shown in Table 3. Regarding cough and rhinorrhea, diarrhea, vomiting, and congestion, there are a high number of professionals and students willing to work with those symptoms but few administrators. Statistical relationships between sore throat and cough and job type are likely due to the relatively larger number of students willing to work with those symptoms. Notably, with the exception of gastrointestinal symptoms, a greater share of students was willing to work with all listed symptoms.

**Table 3:** Counts and percentages of acceptable symptoms by job type Note: Values may not sum to 100%, as participants may have chosen multiple responses. Significant results are bolded. * p ≤ 0.05, ** p ≤ 0.01

Symptoms	Professional	Admin.	Student
Fever, aches, and chills (FAC)	26 (33%)	3 (13%)	12 (43%)
FAC w/ cough	15 (19%)	5 (22%)	11 (39%)
FAC w/ sore throat	21 (26%)	5 (22%)	13 (46%)
FAC w/ diarrhea	15 (19%)	5 (22%)	8 (29%)
FAC w/ vomiting	9 (11%)	3 (13%)	6 (21%)
Respiratory symptoms	34 (43%)	6 (26%)	15 (54%)
Gastrointestinal symptoms	27 (34%)	2 (9%)	9 (32%)
Fever only	20 (25%)	6 (26%)	14 (50%)
Sore throat^*^	49 (61%)	9 (39%)	22 (79%)
Cough^*^	44 (55%)	11 (49%)	23 (82%)
Cough and rhinorrhea^**^	41 (51%)	7 (30%)	21 (75%)
Diarrhea^**^	31 (39%)	3 (13%)	16 (57%)
Vomiting^*^	17 (21%)	1 (4%)	10 (36%)
Congestion^**^	48 (60%)	9 (39%)	23 (82%)
None	9 (11%)	6 (26%)	0 (0%)

A large majority (n = 127, 97%) of respondents believed that working while sick could put patients at risk. Similarly, 118 respondents (90.1%) indicated that if they saw a colleague working while sick, they would report the incident. In terms of preventative behaviors, most respondents washed their hands more than usual (n = 122), covered their cough (n = 117), and wore a mask (n = 110). Finally, we found significant differences based on job type for wearing a mask (p = 0.029), minimizing contact with patients (p = 0.036), and avoiding immunocompromised individuals (p = 0.004). Counts are shown in Table 4. These relationships with job types are likely driven by relatively low numbers of administrative staff willing to take these measures. It should be pointed out, however, that such staff may rarely encounter patients. Also notable is the high percentage of students taking these precautions, relative to healthcare professionals.

**Table 4:** Counts and percentages of precautions by job type Note: Values may not sum to 100%, as participants may have chosen multiple responses. Significant results are bolded. * p ≤ 0.05, ** p ≤ 0.01

Precautions	Professional	Admin.	Student
Wear mask^*^	67 (84%)	14 (61%)	25 (89%)
Wear gloves	34 (43%)	8 (35%)	12 (43%)
Minimize patient contact^*^	49 (61%)	10 (43%)	22 (79%)
Minimize contact w/ colleagues	45 (56%)	13 (57%)	20 (71%)
Cover cough	67 (84%)	19 (83%)	27 (96%)
Avoid immunocompromised patients^**^	40 (50%)	5 (22%)	19 (68%)
Take medication	47 (59%)	12 (52%)	20 (71%)
Other	3 (4%)	1 (4%)	0 (0%)

## Discussion

We show rates of presenteeism among healthcare workers in Jalisco, Mexico that are comparable to rates in other, wealthier nations (67.5% for healthcare professionals compared to ranges of 37-97% reported in other studies [5]), with no statistically significant differences between job types. A large majority (97%) of our sample believed that working while sick puts patients at risk, which is also comparable to a sample from the United States (95%) [7]. Our results indicate that HCW presenteeism is a problem outside of the global north and that most HCWs continue to work while sick, despite the acknowledged risk to patients. Healthcare centers must decisively address this issue to avoid nosocomial outbreaks such as the cluster of influenza A on an oncology ward that led to increased expenditures and a decrease in the quality and quantity of care provided to patients [6]. This is especially so, given that our data were collected during the early stages of the COVID-19 pandemic in Jalisco.

Healthcare workers in our sample were more likely to report working with congestion, sore throat, and cough, which are non-specific symptoms that could indicate multiple conditions, from an ILI to seasonal allergies. This is similar to results from Tartari et al.’s survey, where a high percentage of both HCWs and non-HCWs would work with minor symptoms, including cough [8]. In contrast, Szymczak and colleagues found that most respondents would work while suffering respiratory symptoms (56%) and diarrhea (30%) [7]. The differences in acceptable symptoms between our sample and Szymczak’s may be due to increased symptom vigilance because of the emergence of the COVID-19 pandemic. Revisiting this question after COVID-19 is controlled will allow us to determine whether this is the case, or whether these differences are due to other factors in Mexican healthcare centers. Notably, we found that medical students were generally more likely to work with any symptom (Table 3), though this did not always reach statistical significance. For instance, 54% of students would work with respiratory symptoms, which is very similar to previous results, compared to 43% of medical professionals [7]. However, we also found that students were more likely to take precautions as compared to other healthcare professionals. Exploring the heterogeneity of acceptable symptoms, precautions taken, and presenteeism in general based on job type is an important step toward developing targeted guidance toward reducing infectious disease presenteeism.

As discussed above, there are myriad reasons for infectious disease presenteeism in HCWs, with “sense of duty,” “not wanting to let patients/colleagues down,” staffing concerns, and concerns about being ostracized as predominant concerns in two studies [6,7]. Our results follow this pattern. Not wanting to let patients and colleagues down, concerns about continuity of care, and staffing concerns were the major reasons for presenteeism in our sample. On the other hand, fear of ostracism by colleagues was among the least important reasons. Changes in safety guidelines due to COVID-19 may be responsible for this finding. Because of the pandemic, more attention may have been paid to presenteeism, along with an increase in negative attitudes toward the practice, and hence less tacit encouragement from colleagues. This may also reflect a different culture of presenteeism in Mexican contexts, where presenteeism is not tacitly encouraged by colleagues in general. However, additional research is needed to determine if this is the case. Tartari and colleagues suggested that concerns about staffing and sick leave would be important drivers of presenteeism in lower- and middle-income countries since their healthcare infrastructure is often less supported than those of high-income countries [8]. While we did find that staffing concerns were important (particularly for healthcare professionals compared to administrative staff), insufficient leave was not a major concern. Contrary to Tartari’s suggestion, it seems that staffing concerns may be ubiquitous in healthcare settings. Another important consideration for Mexican HCWs is the possibility of not receiving payment for days they do not work. In Mexico, all workers who feel sick should visit a laboral physician, who checks the severity of symptoms and provides a letter of temporary incapacity allowing the worker to stay at home, as well as any necessary medications. Without this letter, the worker must present to work, even if they feel sick. Otherwise, they will not receive that day’s (or days’) salary. Some HCWs may prefer not to go to a laboral physician with relatively minor infectious symptoms because they know - or believe - the physician would not provide a letter of incapacity. This provides another barrier that can tacitly maintain presenteeism in this context. Policies countering presenteeism should encourage laboral physicians to promote work absences in cases of confirmed or suspected contagious diseases.

Increased handwashing, covering coughs, and wearing a mask were the most commonly listed protective measures taken by professionals, administrators, and students in our sample. Protective measures related to presenteeism are relatively understudied, although Mossad and colleagues found a significant difference in mask-wearing between HCWs attending transplant patients (76%) and internal medicine HCWs (44%) [11]. In general, we find a high adherence to protective measures in healthcare professionals and students. While adherence among administrative staff is generally lower, it is important to note that these individuals may not often encounter patients due to their job responsibilities. Mask-wearing and covering coughs are well represented among administrators, which should help to minimize transmission risk. The high adherence to these protective measures may also reflect concerns about COVID-19. In addition to maintaining these protective behaviors at a minimum, regular and universal symptom screening combined with a referral for testing and treatment may also help to both isolate potentially infectious HCWs from patients and coworkers and monitor levels of presenteeism in healthcare settings [12].

Our results should be considered against the study limitations. Participants were not randomly selected which may have introduced some degree of sample bias in the study and prevented us from generalizing to all HCWs in Jalisco. Our results are also not reflective of HCWs in other Mexican states. Furthermore, our data is self-reported, and we cannot verify the veracity of participant responses. This could introduce the possibility of social desirability bias in responses. However, the survey was completely anonymous and presenteeism rates were largely similar to those from other studies and national/cultural contexts, suggesting that any such bias is minimal or comparable to bias present in other studies. Finally, data were collected during the early stages of the COVID-19 pandemic in Jalisco when healthcare workers shifted to reduced in-person schedules and greater involvement in telemedicine. As noted above, the survey questions asked about behaviors in the past year or in general; this shift in work patterns is therefore not likely to have affected results. Rates of presenteeism in our sample are very similar to pre-COVID rates as well, further supporting the comparability of our results. While the drivers of presenteeism reported by our sample may have changed due to COVID-19, factors are very similar to other studies published well before COVID-19. Nevertheless, repeating this study when COVID-19 is controlled will clarify the degree to which practices were affected by the pandemic and changing work modalities.

## Conclusions

In summation, we find rates of infectious disease presenteeism in Jalisco HCWs that fit with reported ranges in other nations despite the large majority of HCWs acknowledging that such practices put patients at risk. Such trends create conditions for nosocomial outbreaks of infectious disease and should be addressed at multiple levels, including at the health care center and in public health messaging.
